# On Anomalous Symptoms in the Organs of Speech, and in the Circulating System

**Published:** 1815-11

**Authors:** 


*For the London Medical and Physical Journal.*

### *On Anomalous Symptoms in the Organs of Speech, and in the Circulating System;* by S. J. E.

DETERRED, by reasons which would not much ornament the pages of your valuable Journal, from making publicly known my address; I sincerely hope the profession will not, on that account, conceive the following case as unworthy of their attention; although the disease occurs so frequently, yet, there is a strong suspicion dwells with me, that the cause of this impediment might be removed.

A young man whom I have now under my care to instruct in the medical profession, between eighteen and nineteen years old, rather plethoric, of a good family, possessing excellent abilities, who has always been in the habit of taking proper exercise, is now labouring under a severe hesitation in his speech. When only four years old, he fell from a window six feet high, to the stone ground, and pitched on the ossa parietalia, which induced a severe pain in his head for two hours, and then subsided. In two months after, the difficulty of speaking began to affect him; no powerful means were tried to prevent it until he was eight years old, when a clergyman, by relating the consequences, if he allowed it to continue, to him, and never suffering him to discourse but when he spoke distinctly, so far recovered him as to enable him to converse freely. Near half a year afterwards, the impediment returned, which was a good deal abated by the indefatigable perseverance of his tutor when at school, who, after some time, by making him read aloud on the open forest, in the morning air, &c. enabled him to pronounce every syllable with ease. But it is the last paroxysm of his complaint to which I must particularly refer my readers; it commenced soon after an unfortunate occurrence had happened between him and his most intimate friend, which not only produced a furious quarrel, but entirely dissolved their friendship (now about two years ago); it occasioned a severe nervous affection, which made him extremely timid at being introduced into company; he could not bear the least fatigue, was hurried at every trivial event which did not exactly correspond with his wishes, and had many other nervous symptoms. He always had an increase of circulation a short time before he intended to speak, which obliged him to retain the air in his lungs instead of forcing it out, and, while trying to articulate, would sometimes continue to force his words out, while his breath was confined, the thorax expanded, and the circulation impeded, until he appeared as if going into slight convulsions. The disease has been increasing increasing ever since; he has now palpitation; the circulation is generally quick; and his heart will expand sometimes with so great a force that it may be plainly heard for ten minutes at once, at other times very little, and the pulse sometimes intermits; he has slight attacks of rheumatismus chronicus in the deltoid muscles of his arms, often extending part of the way along the biceps flexor cubiti; he has now a very great want of confidence in himself, and sometimes carries it to that excess which obliges him to be silent a whole day together. His general health has been, and is at present, good. Before he took any thing to counteract the effects of the complaint upon his system, the difficulty of speaking brought on a sensation of obtuse pain in the region of the heart and lungs, which subsided soon after the action of the muscles was ended.

I have lately applied a blister on his left side, where the pulsation of the heart is felt, and given him the digitalis purpurea in doses of a grain night and morning; he has, likewise, frequently bathed in a cold bath, at six o'clock in the morning, with considerable advantage, adding vigour to his constitution, but still he has a total want of assurance.

Is this disease owing to nervous affection? or, is there a malformation of the heart or arteries?

Any gentleman taking the case into consideration, and making known his ideas on it, and, likewise, the method he would advise to palliate or remove the symptoms, through the medium of your Journal, as soon as convenient, will confer a favour, which shall in some way be returned by relating the effect his plan (if approved of) has on the patient.

*September* 13, 1815.

