# Errata

**Published:** 1815-12

**Authors:** 


ERRATA.

Page 363, line 41, for *affected* read *effected.*

—— 364, —— 22, for *unobserved* read *unobscured.*

—— 365, —— 39, for *preparing* read *proposing.*

